# (The shift to) online delivery of a rural faculty development program in research skills: lessons learned

**DOI:** 10.1186/s12875-022-01943-0

**Published:** 2022-12-24

**Authors:** Shabnam Asghari, Jonathan Price, Nahid Rahimipour Anaraki, Hensley Hubert Mariathas, Cheri Bethune, Wendy Graham, Andrew Graham

**Affiliations:** 1 Center for Rural Health Studies, Faculty of Medicine Building, Memorial University of Newfoundland, 300 Prince Philip Drive, St. John’s, NL Canada; 2 Center for Rural Health Studies, Discipline of Family Medicine, Faculty of Medicine Building, Memorial University of Newfoundland, 300 Prince Philip Drive, St. John’s, NL Canada; 3 Discipline of Family Medicine, Faculty of Medicine Building, Memorial University of Newfoundland, 300 Prince Philip Drive, St. John’s, NL Canada; 4 Discipline of Family Medicine, Memorial University of Newfoundland, PO Box 250, Port aux Basques, NL Canada

**Keywords:** Rural research, Faculty development, Research skills, Program delivery, Program administration, Virtual delivery model, Capacity building, COVID-19

## Abstract

**Background:**

While rural physicians are the ideal candidates to investigate health and healthcare issues in rural communities, they often lack the required skills, competencies, and resources. As a result, research skills development programs are crucial to help ensure communities receive the quality of care they deserve. Memorial University of Newfoundland created a research skills development program called 6for6 to empower and enable rural physicians to research solutions to community-specific health needs. 6for6 program delivery was exclusively in-person until 2019. However, with limitations introduced due to the COVID-19 pandemic, organizations around the globe needed to respond quickly. As we work to return to a post-pandemic environment, program administrators and educators worldwide are unsure whether to retain or remove the changes made to programs to adapt to the pandemic restrictions. Therefore, this work addresses the impact of the online delivery model in two areas: 1) attainment of competencies (specifically research skills, knowledge, and attitudes); and 2) participant experiences, defined as the ease of attendance, the capacity to interact with team members and peers, and challenges or barriers associated with navigating program resources.

**Methods:**

We compared the effect of an online delivery model pivoted to adapt pandemic restrictions with the original model (primarily face-to-face) on the acquisition of learning competencies and participant experience using a mixed-methods study. Various data collection methods, such as a pre-post program survey, post-program focus group, and structured observation, were utilized.

**Results:**

From 2014 to 2021, 35 physicians attended the program (30 face-to-face and five online). The Wilcoxon-sign-rank test did not show any significant differences in the participants’ median change of research competency scores who attended face-to-face and online learning, respectively: knowledge (32.6, 26.8), attitudes (3.8, 3.5), and skills (32.4, 20.0). Flexibility and accessibility were key aspects of participants’ experiences during the online model. Comparison with previous years demonstrated no significant challenges with the virtual delivery model, yet participants struggled with mentorship challenges and learning-life balance.

**Conclusions:**

Although presenting some unique challenges, the online model did not negatively affect learner competencies. Likewise, it provided opportunities for rural physicians to attend learning sessions and interact with experts and peers while remaining in their communities.

## Introduction

Rural physicians are well-positioned and often motivated to conduct community-based research [1, 2] but face many barriers, including inadequate research training [3, 4]. Therefore, professional development programs in research skills provide rural physicians with the appropriate tools to conduct quality research in complex rural environments [4]. With this in mind, a team at the Discipline of Family Medicine, Faculty of Medicine at Memorial University (Memorial) in St. John’s, Newfoundland and Labrador, Canada, designed and implemented a research skills development program to facilitate rural health research [5]. The program provides rural physicians with dedicated training and mentorship in academic research and writing. Kern and colleagues’ 6-step curriculum development approach for medical education was used to ground the design and development of the program’s framework and identify goals, learning objectives, curriculum content, and instructional strategies, as previously described [6]. We delivered the program in-person until 2019 and effectively facilitated the development of research competencies of rural physician participants [7].

With the onset of the global COVID-19 pandemic in early 2020, organizations worldwide implemented various safeguards. As a result, we quickly pivoted the program at Memorial to an online-only delivery model for 2020-21. Now, more than a year after the start of the pandemic, we are undertaking a comparison of two delivery models (primarily face-to-face vs. entirely online) to help determine the ideal future delivery model. This work will provide a foundation for other organizations that are preparing to return to a new, post-pandemic work environment.

## Aims and objectives

Our purpose was to assess the impact of the online delivery model in two areas: 1) attainment of competencies (specifically research skills, knowledge, and attitudes); and 2) participant experiences, defined as the ease of attendance, the capacity to interact with team members and peers, and challenges or barriers associated with navigating program resources. To explore these questions, we undertook a mixed-methods study approved by the provincial Health Research Ethics Board.

### Who we are

The team consisted of the core program team (research methodologists, faculty development experts, medical education experts, rural clinicians, and a program coordinator) and the teaching team (subject-matter experts, program mentors, and guest lecturers [e.g., past rural physician participants, national and international experts, and senior members of healthcare organizations]). The core program team was involved in all aspects of program conceptualization, design, and analyses, whereas the teaching team was focused solely on program delivery. Program participants were individually paired with expert researchers, or mentors, to help them with the curriculum content and with integrating the content into their research plans. The participants regularly met with their mentors to develop their projects, and in turn, develop a productive working relationship. Mentors were carefully selected, with consideration given to factors such as expertise, time availability, and experience. Since 2022, program alumni have acted as mentors for new participants, which highlights the full-circle nature of this program. All mentors are provided mentorship support through expert-guided face-to-face discussions with the core team, resources like coaching strategies, and a sample timeline for meeting program outcomes.

### “6for6”: a research skills development program

The research skills development program, 6for6, is developed around an adult learner-centered approach using a blended learning model and is tailored to the needs of physicians practicing in rural areas of Canada. Six participants per year gain various research competencies and enhance their writing skills while developing their own research idea (the capstone project).

The program utilizes both synchronous (instructor-led) and asynchronous (self-paced) content. The synchronous content comprises six structured, face-to-face sessions delivered at the main Memorial campus in St. John’s. The asynchronous content is a number of dedicated self-learning activities and assignments, including readings, tip sheets, workbooks, writing exercises, and online resources.

The team invited various subject-matter experts (e.g., qualitative and quantitative research, ethics, library services, knowledge translation, community engagement, and scholarly writing) to help with each of the six face-to-face sessions. The core team developed the session goals and learning objectives in consultation with subject-matter experts using the anchored delivery model [8]. The program utilizes team teaching, lectures, small discussion groups, class activities, Q&A, and peer learning.

### Pivoting the program online

During the spring, summer, and fall of 2020, the core team quickly pivoted and adapted to pandemic restrictions and so developed an online-only delivery model for the 2020-21 group (delivered over a condensed time frame of seven months from September 2020 to March 2021; reduced from a typical 12-month time frame).

The first step was converting the face-to-face (synchronous) content into an online format. The core team completed this conversion internally. A priority was determining how to incorporate these elements into a virtual model (e.g., participants connecting with their mentor, the librarian, and the program coordinator); this included conducting an environmental scan to determine the best approach for delivering the content online. The team also completed an extensive literature search to identify any related evidence.

The next step was reviewing course content to determine any required changes. The team completed internal and external consultations, including education design experts, subject-matter experts, and 6for6 alumni. Given that the rurally-based participants and team members had varying access to reliable internet, the team sought input from the 2020-21 group to help ensure the best possible experience. The team also mapped each course concept with corresponding modes of delivery to determine the best use of available resources and to maintain the core components of 6for6, including participant interaction and engagement, while working within pandemic restrictions. To assist with this, the team utilized the Capability Assessment Framework to help determine institutional capability for pivoting online [9]. As noted in Table 1 below, while course content remained the same, the mode of delivery of many components changed.

**Table 1:** 6for6 program components when comparing face-to-face and online delivery models

Program Component	Face-to-Face Model	Online-Only Delivery Model
Course Learning Platform	Online platforms including D2L (Desire2Learn)	D2L
Teaching Strategy	In-person lectures and discussion at Memorial’s main campus	Online sessions (with the addition of 6for6 alumni and international colleagues as guest presenters), recorded videos, podcasts, interactive learning activities, real-world case studies, online debates, whiteboard teaching, and learning planning
Communication Medium	Face-to-face (group discussions and one-on-one meetings), email, phone	Online meeting platform (group discussions and one-on-one meetings via WebEx/Zoom), email, phone
Course Content	Exact same course content across both delivery models	
Meetings with Mentors	In-person meetings at Memorial’s main campus	Online meetings
Assignments	Hard copies, oral presentations Oral feedback Flexible with deadline	Online submissions via D2L with restricted deadline and written feedback
Dedicated Research Time	In-person and on campus with office/computer access	Completed during participants’ own time/availability at home
Meetings with Research Support Staff	In-person and on campus with office/computer access	Online meetings
Networking	In-person meetings planned by the team	Self-motivated online
Class Time	Friday + Saturday	

As part of the inaugural online year, we also asked participants about their home technology (e.g., internet connectivity) and familiarity with online programming. Subsequently, we provided training in navigating online platforms such as WebEx and D2L (Desire2Learn), Memorial’s online learning platform, and a toll-free telephone option as a backup. We also recorded online sessions to share with participants via D2L. These online components were additions not previously available when the mode of delivery was primarily face-to-face.

## Methods

### Survey (pre-post program survey)

The pre-and post-program survey measures self-assessed research competency, which is a subjective measure of the relationship between knowledge, attitudes, and skills of an individual that combine to produce results [10]. The categories used by MacLellan [7], define knowledge as “participants’ textbook understanding of research concepts and their ability to recall the information,” attitudes as the degree “to which one views research as valuable and worthwhile,” and skills are participants’ “ability to put research knowledge into practice.” Surveys were developed using a modified Delphi approach to identify and organize a suite of questions, which were later consolidated and carefully customized for our specific audience. As previously described, the survey was pilot tested [10]. Details about the development and validation of the surveys are available via our previous works [6, 7, 10]. Surveys for all cohorts were administered online.

### Post-program focus group

We conducted the post-program focus group as part of the final session with each group from 2014 to 2021. All participants’ (*n* = 35) were encouraged to attend a focus group at the end of the last program session; to date, 35 rural physicians have participated. Two third-party researchers with expertise in qualitative research methods facilitated this session. The focus groups were semi-structured; participants were asked about advantages and disadvantages of the program, suggestions to improve program delivery, and important takeaways, among other questions. Focus groups were held in-person until we were required to pivot to an online format for the 2020-21 cohort (due to the COVID-19 pandemic). This focus group was conducted via Zoom. Participants were asked additional questions about the virtual nature of the program, such as pros and cons related to online delivery. These additional questions were approved by the provincial Health Research Ethics Board.

### Structured observation

Researchers, mentors, and facilitators conducted structured observation during the delivery of each group from 2014 to 2021 and kept detailed field notes. Observation notes include details on barriers or challenges experienced by team members and participants, suggestions for improving the program and participant experiences, and strategies for motivating and encouraging participants. Structured observation was supplemented by focus group data for a more thorough analysis.

### Analysis

We measured self-assessed research competencies (knowledge, attitudes, and skills) using a five-level Likert Scale. We estimated participants’ scores for competencies as total scores obtained by each participant on the questions related to each competency (knowledge, attitudes, and skills) divided by the total possible score for each competency and multiplied by 100. We summarized research competencies using mean, standard deviation (SD), median, and interquartile ranges (IQRs). We performed a Wilcoxon signed-rank test to examine any statistical difference in knowledge, attitudes, and skills before and after the program and any differences between face-to-face and online delivery models using score changes between pre-and post-program. We performed analyses in the R version 4.1.1 (R Core Team, 2020, R Foundation for Statistical Computing, Vienna, Austria) statistical software package to obtain the results. We considered a *p*-value < 0.05 statistically significant.

For qualitative analysis, we utilized a thematic analysis of the post-program focus group and structured observation to extract comprehensive information regarding learner experiences. Extracted observational data in this study was integrated as confirmatory research. In other words, structured observation data was used to corroborate research findings in this study. We transcribed the focus group discussions verbatim, and qualitative data experts undertook the thematic analysis. A researcher with expertise in qualitative analysis described the scope and content of each developed theme. The researcher then established the final themes, compared them with the data and coding set to ensure accuracy, and discussed them with the core team to reach a consensus. The extracted themes from previous cohort focus groups (i.e., in-person model of delivery) associated with research competency, networks, and connections, were considered a basis for analysis of the focus group and structured observations performed using the online delivery model.

## Results

Thirty participants attended the program (six participants per year for five years) during face-to-face delivery. The response rate during this time frame was 96.7% (*n* = 29) for the pre-program survey and 76.7% (*n* = 23) for the post-program survey. Respondents were 56.7% (*n* = 17) female and 43.3% (*n* = 13) male; 83.3% (*n* = 25) family physicians and 16.7% (*n* = 5) other specialists.

During the one year of online delivery, five participants attended the program. There was one drop-out early in the program (*n* = 6). Respondents were 80.0% female and 20.0% male; 80.0% family doctors and 20.0% other specialists.

### Learner competencies

Over the five years of face-to-face delivery, the median score of self-assessed research competency in knowledge, attitudes, and skills changed from 51.7, 89.1, and 49.5 before the program to 83.3, 95.7, and 80.0 after the program. The assessment of paired difference in the areas of knowledge (*p* < 0.001), attitudes (*p* < 0.05), and skills attainment (*p* < 0.001) were statistically significant (see Table 2 below).

**Table 2:** Research competencies pre- and post-program among 6for6 participants during face-to-face delivery between 2014 and 2019 (*n* = 30)

Research Competencies	Pre-Program		Post-Program	
	Mean (SD)	Median (IQR)	Mean (SD)	Median (IQR)
Knowledge	50.0 (12.0)	51.7 (15.0)	85.2 (9.0)	83.3 (13.1) ^**^
Attitudes	87.0 (10.5)	89.1 (16.4)	93.5 (7.0)	95.7 (11.3) ^*^
Skills	50.0 (12.7)	49.5 (17.0)	81.4 (10.2)	80.0 (15.2) ^**^

^*^ *P*-Value for Wilcoxon signed rank test < 0.05

^**^P Value for Wilcoxon signed rank test < 0.001

Only those who responded to both the pre- and post-program surveys (*n* = 23) were considered for the test

For online learning, the median research knowledge, attitudes, and skills scores were 59.7, 81.7, and 63.1 pre-program compared to 80.0, 90.4, and 80.0 post-program. However, the data size did not support statistical significance using an appropriate statistical test (see Table 3).

**Table 3:** Research competencies pre- and post-program among 6for6 participants (*n* = 5) attending online delivery during 2020-21

Research Competencies	Pre-Program		Post-Program	
	Mean (SD)	Median (IQR)	Mean (SD)	Median (IQR)
Knowledge	57.0 (7.9)	59.7 (9.0)	86.3 (9.3)	80.0 (16.5)
Attitudes	82.8 (6.5)	81.7 (8.7)	89.2 (7.2)	90.4 (8.7)
Skills	62.8 (7.3)	63.1 (5.6)	83.4 (7.1)	80.0 (12.8)

No statistical analyses were performed for the above data

As shown in Table 4, the changes in competencies and the Wilcoxon sign rank test did not identify any significant changes in research competencies between the face-to-face and online delivery models.

**Table 4:** Changes in research competencies among 6for6 participants by mode of delivery (face-to-face vs online) between 2014 and 2021

Research Competencies	Face-to-face (***n*** = 23)		Online (***n*** = 5)	
	Mean (SD)	Median (IQR)	Mean (SD)	Median (IQR)
Knowledge	35.1 (14.6)	32.6 (26.6)	29.4 (14.4)	26.8 (14.5)
Attitudes	4.7 (8.0)	3.8 (11.6)	6.4 (6.6)	3.5 (7.0)
Skills	30.7 (11.9)	32.4 (20.3)	20.6 (4.5)	20.0 (7.2)

No statistical significance between face-to face and online delivery using the Wilcoxon sign ranked test. No statistical significance within the face-to-face mode by year of delivery

### Learner experiences

A comparison of the focus group and observation findings from the virtual delivery model and face-to-face model did not show any significant challenges for participants. Regardless of the delivery model (face-to-face or virtual), attendees strongly emphasized the program’s key role in expanding their research competency and networks and connections, providing them with a unique opportunity to incorporate research skills into their daily lives as rural physicians. Analysis of the post-program focus group during the pandemic illustrated several benefits of the online-only delivery model, including the ease of attending the learning sessions (i.e., flexibility and accessibility) and the capacity to interact with team members and peers (i.e., cultivating networks and connections) while also pinpointing some challenges or barriers associated with navigating program resources (e.g.,  learning in a different time zone with a mismatched mentor and learning-life balance).

### Flexibility and accessibility

Due to geographical and professional isolation and a shortage of time and funding, rural physicians constantly struggle with a lack of accessible and contextually-relevant research training programs. 6for6 addresses the gap between research and clinical practice for rural physicians. Nevertheless, face-to-face sessions over six weekends (Friday and Saturday only) during one year demands significant travel for attendees. The virtual version of the program; however, provided flexibility and accessibility for busy rural physicians who may not be able to find time for the program in their busy schedules otherwise:

“*I really liked that it was virtual. I know that this was intended to be an in-person session, but I found that really added a bit of flexibility to it and made it a lot easier to accommodate it. I don’t think I would have been able to attend all of the sessions in-person, if that’s how it went; just with work scheduling stuff … you know, there’s the things that we do together.*”

### Cultivating networks and connections

Attendees have considered the lack of a supportive research system as one of the barriers to conducting research for anyone practicing in rural and remote areas. An undeniable impact of 6for6, other than building research capacity (i.e., knowledge, attitudes, and skills) and productivity (i.e., publications, grants, and conference presentations), is building professional research networks and connections, which includes facilitators, researchers, the program coordinator, mentors, librarians, and colleagues:

“*I’ve lived in [remote areas] for most of my career, right? And so, how to connect was my main preoccupation; that felt like the biggest barrier and I feel like that barrier is gone now. Right, so it’s not necessarily skills but it’s like plugging in and knowing who to ask and how to find them in the end and just feeling like there are fewer barriers.*”

The primary requirement to build this professional circle of support is having rich peer-to-peer and mentor-participant communication. While one might assume that the virtual format may challenge engagement, communications, connections, and weaken supports, participants noted they had a substantial amount of interaction and effective communication with team members under the virtual delivery format:

“*[A]nd I was like quite surprised right off the bat at the intimacy that the virtual still allowed us, you know.*”

Furthermore, participants applauded the cooperation and teamwork among peers during the program’s online delivery. In addition to increasing interactivity and finding comfort in knowing that other people shared in their struggles (e.g., work-life balance), it also helped ensure that participants all remained motivated in their respective projects:

“*We’re all in fairly similar situations we have busy clinical work and family life and otherwise, so you know, knowing that we’re all sort of trying to balance it all and learn from each other has been really great.*”

### Learning in a different time zone with a mismatched Mentor

One of the program goals is to uncover and modify the barriers and difficulties caused by the program curriculum and structure. Even though the majority of participants were satisfied with their mentor-mentee relationship and found it to be a helpful aspect of the program, time zone differences and mismatched mentors were considered challenges. Time zone differences between the participants and their mentors contributed to rescheduled meetings and delays. In addition, one online participant felt that they were not matched with the most appropriate mentor for their project; a concern that was not immediately noticed by the study team due to the online program delivery:

“*I really thought I was coming in and doing a quantitative study. That was my idea, so I was paired with someone qualitative which I think was like both good and bad, right?...I was like trying to figure out how to do my study and like, I think probably a month or two of just like really trying to jam my square peg into a round hole and I think, partly because I really felt like there was an expectation that I was going to do qualitative research because I’m paired with a qualitative research partner”.*

The mentor-mentee selection is based on the participant’s research question and the mentor’s expertise. We strive to make the best selection possible for both the mentor and mentee; however, communication style, availability, and clinical background, among many other factors, can affect this relationship. When the 6for6 program was delivered exclusively in-person, the core team was able to monitor for these factors and mediate the mentor-mentee relationship, as required. Mentorship issues can arise in both the in-person and online delivery models; however, it is much more difficult to monitor and mitigate these issues during online program delivery.

### Learning-life balance

Workload and time frame have been regularly monitored, evaluated, and reframed during the in-person model of the program. Although the online delivery model provides participants with the opportunity to attend the classes while being at home, attendees not only have to complete all the required asynchronous content and assignments of the program, but also meet commitments outside of the program. This comes with feeling overwhelmed by the course commitments while trying to balance home and work responsibilities.

“*[W]here sometimes the assignments or information was coming out really close to when the sessions were going to be, or the deadlines; I found that pretty tricky, for you know as an adult learner who’s like trying to balance other commitments.*”

The respective challenge is unique to the online delivery model, as face-to-face delivery included allotted time for participants to work on 6for6 material. In addition, participants who attended the face-to-face program were able to step away from other obligations for the weekend of the session and focus solely on their research projects:

“*Things that I think made a difference for me … creating time and space. My clinic is closed, I’m here, and I’ve never been more productive around scholarly work than those two or three days that I’m here because I’m not distracted and people are priming my brain.”*

*“Get out of your space, and … you think differently, work differently, have different experiences, I’m a different practitioner now because of that.”*

Unlike participants who completed the in-person program, participants in the online-only group were simultaneously dealing with their professional obligations and challenges (e.g., changing clinical arrangements) and home-related responsibilities (e.g., meeting household errands and providing childcare due to closure of schools), which at times interfered with their course engagement and productivity. In-person program delivery provides participants the opportunity to have dedicated time away from their other responsibilities to focus on their research. We also noted that time zone differences resulted in less engagement by participants, particularly due to interweaving with family responsibilities. Regardless, any additional educational activities will cause a busy clinician to become even busier, whether delivered online or in-person.

## Discussion

Many organizations will be seeking answers as they prepare to return to a new, post-pandemic work environment. So, what is the optimal model of delivery moving forward for supporting participant needs? Is it a return to a traditional in-person program delivery model, continuing with an online model, or a combination of both? Of course, if we remain restricted by the pandemic impacts, there is no option but to remain online. However, if lifted, it becomes imperative to determine if virtual delivery models are as effective and acceptable for our participants and program faculty as a face-to-face modality.

Our assessment suggests that the online delivery model, while presenting its own unique challenges for both team members and participants, did not negatively impact the attainment of self-reported research competencies, and we were able to support the learning needs of the rural participants. These results echo the findings of existing literature, noting a lack of material difference between in-person and online delivery models [11, 12] while reinforcing the benefit of mixing synchronous and asynchronous content when engaging participants online [13, 14]. O’Doherty et al. identified a number of barriers and solutions for the development and implementation of an online learning program, which reiterates our findings. For example, a lack of time was identified as a barrier due to the ever-increasing demands placed on both students and educators. The authors’ solution to this barrier is the notion that through using online learning techniques, more time will become available as the process is streamlined [15].

Our findings should be interpreted in light of their limitations. It should be noted that this study was limited to just one year of data on the online-only delivery model, which was rapidly assembled due to the uncertain pandemic regulations and consequently delivered over a condensed time frame. Ideally, the curriculum would have continued for 12 months with adequate preparation time. Although our assessment did not show any difference in self-assessed research competencies by delivery model, a non-inferiority design study would better support whether face-to-face and online delivery are equivalent. We also did not have sufficient data to support statistical analysis for assessing competencies before and after online delivery.

Admittedly, the online curriculum included some deficiencies (e.g., lack of time to complete asynchronous material), which would not have been a significant issue in a non-pandemic year. Furthermore, face-to-face delivery included time allocation for self-paced research activities while the participants were on campus. In addition, the pandemic conditions may have influenced participant satisfaction with the online delivery model (i.e., no other options during the pandemic).

One of the goals of our rural research capacity building model is to empower rural practitioners to be change-makers in their communities by investigating contextually relevant health issues [16]. We continually evaluate the application and impact of the program in improving rural and remote healthcare practices and policies. Details on the evaluation and short-term and long-term outcomes of the program before the pandemic are available via our previous works [10, 16, 17] and the alumni’s work on improving rural and remote healthcare practice [18–20]. The present study aimed to examine the effect of the shift to online delivery during the pandemic on the attainment of competencies and participant experiences of the training. Although we regularly collect data on the program’s impact in improving rural and remote healthcare practices and policies, it may take several years after the pandemic to have comparable data to reflect on this impact. Future studies will explore the impact of the program’s online delivery on alumni’s work and improving rural healthcare practice. For instance, do participants achieve as many measurable outputs (e.g., dissemination activities) compared to face-to-face groups? Moreover, what is the impact of an online delivery model on rural research network building and collaboration?

## Conclusion

As program developers and organizers prepare for a new normal, ideally with fewer restrictions, these questions will become increasingly relevant to ensure the best program for participants while working within the boundaries of a post-pandemic workplace. In our case, one option would be to utilize a hybrid model of delivery, including the best aspects of both the online model and the original face-to-face model. Among other benefits, this would allow our participants greater flexibility to stay in their communities when they are most needed while still offering some advantages of a face-to-face model.

While this study focuses on rural physicians participating in a research skills development program in Canada, the results will have global applicability for any organization seeking to determine an optimal delivery model for distributed clinicians and, ultimately, will also help ensure positive participant outcomes in many sectors.

## Data Availability

The datasets used and/or analysed during the current study are available from the corresponding author on reasonable request.

## Abbreviations

D2L: Desire2Learn
SD: Standard deviation
IQR: Interquartile range
